# High Concentrations of Aspartame Induce Pro-Angiogenic Effects in Ovo and Cytotoxic Effects in HT-29 Human Colorectal Carcinoma Cells

**DOI:** 10.3390/nu12123600

**Published:** 2020-11-24

**Authors:** Anca Laura Maghiari, Dorina Coricovac, Iulia Andreea Pinzaru, Ioana Gabriela Macașoi, Iasmina Marcovici, Sebastian Simu, Dan Navolan, Cristina Dehelean

**Affiliations:** 1 Faculty of Medicine, “Victor Babeș” University of Medicine and Pharmacy Timisoara, Eftimie Murgu Square No. 2, RO-300041 Timișoara, Romania; boscu.anca@umft.ro (A.L.M.); navolan.dan@umft.ro (D.N.); 2 Faculty of Pharmacy, “Victor Babeș” University of Medicine and Pharmacy Timisoara, Eftimie Murgu Square No. 2, RO-300041 Timișoara, Romania; dorinacoricovac@umft.ro (D.C.); macasoi.ioana@umft.ro (I.G.M.); iasmina.marcovici@umft.ro (I.M.); simu.sebastian@umft.ro (S.S.); cadehelean@umft.ro (C.D.)

**Keywords:** aspartame, colorectal carcinoma, angiogenesis, HT-29 cells, CAM assay, cytotoxicity

## Abstract

Aspartame (ASP), an artificial sweetener abundantly consumed in recent years in an array of dietary products, has raised some concerns in terms of toxicity, and it was even suggested a link with the risk of carcinogenesis (colorectal cancer), though the present scientific data are rather inconclusive. This study aims at investigating the potential role of aspartame in colorectal cancer by suggesting two experimental approaches: (i) an in vitro cytotoxicity screening in HT-29 human colorectal carcinoma cells based on cell viability (Alamar blue assay), cell morphology and cell migration (scratch assay) assessment and (ii) an in ovo evaluation in terms of angiogenic and irritant potential by means of the chorioallantoic membrane method (CAM). The in vitro results showed a dose-dependent cytotoxic effect, with a significant decrease of viable cells at the highest concentrations tested (15, 30 and 50 mM) and morphological cellular changes. In ovo, aspartame (15 and 30 mM) proved to have a pro-angiogenic effect and a weak irritant potential at the vascular level. These data suggest new directions of research regarding aspartame’s role in colorectal cancer.

## 1. Introduction

Simple sugars have always been an inseparable and important component of the human diet. Nonetheless, in the 19th century, sucrose, which became a common consumer product, replaced the sugars provided by fruits and honey [1]. Due to the recent ‘sweetening of the world’s diet’, there has been a dramatic rise in the consumption of added sugar [2]. There is ample evidence linking the consumption of sugar-sweetened food products to adverse effects such as long-term weight gain, obesity and prevalence of metabolic and cardiovascular diseases [1,3]. Furthermore, it has been suggested that sugar leads to a drug-like addiction, its habit-forming character being comparable to cocaine, nicotine, alcohol, tobacco, and caffeine [2]. The recent growth of health awareness among populations has led to the replacement of sugar with natural and artificial sweeteners [3] that belong to an important class of additives able to increase the effect of sugar in taste [3] and also to enhance food flavor, while contributing very little to energy intake [4]. Moreover, several therapeutic indications for sugar substitutes, such as weight loss, dental care, and the control of glycemic levels in diabetes, have been described. Regarding their health risks, the topic is still a matter of debate [3].

Aspartame (ASP), 1-methyl N-l-[alpha]-aspartyl-l-phenylalanine (C_14_H_18_N_2_O_5_), is an artificial sweetener that was accidentally discovered in 1965 by Schlatter JM during its attempts to discover new drugs for ulcer treatment [5]. The uniqueness of ASP is derived from its clean sugar-like taste and potent sweetness which is 200 to 300 times higher compared to sucrose [6]. Furthermore, aspartame was reported to possess antipyretic, analgesic, and anti-inflammatory action [3]. Even though ASP has been a diet component for over 30 years [7], there continues to be great interest in researching its toxicity, particularly pertaining to carcinogenic risk [8]. Several studies suggest a possible risk of cancer development associated with the intake of aspartame. However, these findings do not correlate with findings in humans [9], and the implications of ASP in carcinogenesis remain controversial.

Colorectal cancer (CRC) is one of the leading causes of death worldwide, with an incidence that is growing at an alarming rate and occupies the third place worldwide in terms of diagnostic frequency [10,11,12]. Even though surgery is the main procedure in patients when diagnosed early [11], during the late and metastatic stages of CRC, chemotherapy remains the core of cancer treatment [10]. Environmental and genetic factors play a major role in the pathogenesis of colon cancer. The role of nutrition in colon cancer has been studied extensively, explaining a causal and protective role in the development of colon cancer [13,14]. The erroneous dietary habits (e.g., a typical Western diet)—namely, the intake of products with high content of saturated fats, sugar, and artificial sweeteners; and a low consumption of vitamins, calcium, and fibers—represent a strong determinant for the development of colorectal carcinoma [15,16]. The scientific reports that indicate a link between the consumption of artificial sweeteners and colorectal cancer occurrence are rather scarce and contradictory, with several sustaining a direct correlation between the two [16,17,18], whereas more recent studies contest a potential connection [7,19]. Based on these data, it could be stated that the role of aspartame in CRC is still inconclusive and further studies are required to clarify this dilemma.

A reliable indicator of poor prognosis in CRC is the augmented density of blood vessels (angiogenesis) within the tumor tissue. Angiogenesis is a central player in CRC; most of the processes that occur in this pathology (growth of primary tumor and metastasis) are strongly dependent on new blood vessel formation [12]. According to a previous study [20], aspartame exerts a stimulatory effect on angiogenesis in both in vitro and in vivo conditions. These data offer a new perspective on aspartame’s role in carcinogenesis, yet more studies are needed to define this aspect.

Given the scientific background, this paper aims to investigate the potential role of the artificial sugar substituent aspartame in colorectal cancer by applying two experimental approaches: (i) an in vitro screening on HT-29 human colorectal carcinoma cells based on cell viability, cell morphology, and migration assessment and (ii) an in ovo evaluation in terms of angiogenic and irritant potential.

## 2. Materials and Methods

### 2.1. Cell Culture and Reagents

The human colorectal carcinoma cell line HT-29 (ATCC^®^ HTB-38™), purchased from ATCC (American Type Cell Collection), was cultured in specific medium: McCoy’s 5a modified medium (ATCC^®^ 30-2007™) completed with 10% fetal bovine serum (FBS, Gibco) and 1% of antibiotic mixture (penicillin and streptomycin, Sigma Aldrich, Merck KGaA Darmstadt, Germany). Standard conditions were implemented for cell culturing: a temperature of 37 °C and 5% CO_2_ in a humidified incubator.

Aspartame powder, penicillin and streptomycin mixture, phosphate-buffered saline (PBS), trypsin EDTA, Trypan blue, and Alamar blue (resazurin) were acquired from Sigma Aldrich, Merck KGaA. The cell culture specific media—McCoy’s 5a Medium Modified (ATCC^®^ 30-2007™) and fetal bovine serum (FBS, Gibco)—were purchased from ATCC and Thermo Fisher Scientific, Inc., Waltham, MA, USA, respectively. All the reagents were of analytical standard purity and were applied according to the manufacturers’ recommendations.

### 2.2. Cell Viability Assay

The cell viability assessment was performed using Alamar blue test. The HT-29 cells were seeded in 96-well plates at 1 × 10^4^ cells/well/200 μL culture medium and allowed to grow for 24–48 h (until the optimal confluence was reached). Treatment of cells with different concentrations of aspartame (ASP) solution in PBS, of 0.1, 0.25, 0.5, 1, 3, 6, 15, 30, or 50 mM, was followed by a 72 h incubation. A volume of 20 μL/well of Alamar blue reagent was added into each well and the plate was incubated for 3 h at 37 °C. The values of the absorbance were measured at 570 and 600 nm using a xMark™ Microplate spectrophotometer (Bio-Rad). The results were expressed as percentage of viable cells (%) and were calculated using the following formula [21]:

(1) $$\begin{array}{l} \mathrm{Viable}\;\mathrm{cells}\;\left(\%\right)\\ =\frac{\left(\varepsilon_{\mathrm{OX}}\right)\lambda_{2\;}A\lambda_{1}-\left(\varepsilon_{\mathrm{OX}}\right)\lambda_{1}A\lambda_{2}\;\mathrm{of}\;\mathrm{test}\;\mathrm{agent}\;\mathrm{dilution}}{\left(\varepsilon_{\mathrm{OX}}\right)\lambda_{2}\;A^{{}^{\circ}}\lambda_{1}-\left(\varepsilon_{\mathrm{OX}}\right)\lambda_{1}A^{{}^{\circ}}\lambda_{2}\;\mathrm{of}\;\mathrm{untreated}\;\mathrm{positive}\;\mathrm{growth}\;\mathrm{control}}\times 100 \end{array}$$

where:

ε_OX_ = molar extinction coefficient of Alamar blue’s oxidized form (BLUE)
A = absorbance of test wells
A° = absorbance of positive growth control well (cells without tested compounds)
λ_1_ = 570 nm and *λ*_2_ = 600 nm.

### 2.3. Cell Morphology

As part of the toxicological profile of aspartame, a microscopic evaluation of the cells’ morphology and shape was performed to notice the potential changes induced by aspartame treatment. The cells were observed under bright field illumination and photographed at 72 h post-treatment with aspartame solubilized in PBS, at 0.1, 0.25, 0.5, 1, 3, 6, 15, 30, and 50 mM, using an Olympus IX73 inverted microscope (Olympus, Tokyo, Japan). The photos were analyzed with the cellSens Dimensions v.1.8. Software (Olympus).

### 2.4. Cell Migration

A wound healing assay—scratch assay—was conducted to assess the effect of aspartame solution on the migratory capacity of HT-29 cells. The experimental protocol consisted of the following steps: (1) 2 × 10^5^ HT-29 cells/well/1.5 mL culture medium were cultured in 12-well plates; (2) the scratches were drawn using a sterile pipette tip; (3) fresh culture medium with test compound (aspartame solution in PBS: 1; 3 and 6 mM) was added; (4) culture was incubated for 24 h. The cells were pictured at 0 and 24 h using the DP74 camera integrated within the Olympus IX73 inverted microscope (Olympus, Tokyo, Japan). The scratch widths were measured at 0 and 24 h using CellSense Dimension 1.17. Software (Olympus). The scratch closure/migration rate (%) was calculated with the following formula:

(2) $$\mathrm{Scratch}\;\mathrm{closure}\;\%=\frac{A_{t=0h}-A_{t=24h}}{A_{t=0h}}\times 100$$

where:

A_t=0h_ is the width of the wound measured at 0 h.
A_t=24h_ is the width of the wound measured after 24 h [22,23].

### 2.5. Chorioallantoic Membrane Assay (CAM)

To perform the CAM method, white embryonated chicken eggs (*Gallus gallus domesticus*) were used. The eggs were purchased from local providers. The protocol employed was conducted according to the literature [24] with slight changes: (1) the eggs were disinfected with 70% ethanol and incubated in standard conditions (constant temperature of 37 °C and 50% humidity); (2) on the third day of incubation, 5 mL of albumen was removed to observe the blood vessels; (3) on the fourth day, a window was cut in the upper part and was sealed with adhesive tape; and (4) the eggs were incubated until the day of the experiment. The observation of the blood vessels was performed microscopically, daily, with the help of the Discovery 8 Stereomiscroscope microscope (Zeiss, Göttingen, Germany). The images were taken using an Axio CAM 105 color Zeiss digital camera and processed with Zeiss ZEN software: Image J and GIMP.

### 2.6. Normal Angiogenesis Assessment of the Chorioallantoic Membrane

This type of study is suitable both for verifying the tolerability of ASP and for observing its effect on normal angiogenesis. The evaluation of ASP effect on angiogenesis began on the seventh day of incubation and was performed for 4 days. During this period, the blood vessels showed a rapid rate of development, similar to that of the tumor process. Plastic rings of 3 mm diameter were placed on the CAM. Three different concentrations of ASP (6, 15, and 30 mM) were tested after addition inside the ring, at 10 µL/egg. PBS, the solvent used in the preparation of ASP solutions, was used as a control by applying the same three concentrations as for ASP (6, 15, and 30 mM).

### 2.7. Hen’s Egg Test on Chorioallantoic Membrane (HET-CAM) Assay

The hen’s egg test on chorioallantoic membrane (HET-CAM) was used to test the biocompatibility and irritant potential of ASP. For each concentration tested, five eggs were used, prepared as described above. Two concentrations of ASP (15 and 30 mM) and PBS (control) were tested, applied in a volume of 600 µL.

As a positive control, a solution of 1% sodium dodecyl sulfate (SDS) in water was used. The following effects on blood vessels were observed for 5 min: hemorrhage—H, vessel lysis—L, and coagulation—C. The biocompatibility and irritant potential of ASP was determined by the analytical method, which involves the calculation of the irritation score (IS) using the following formula [25]:

(3) $$\mathrm{IS}=5\times \frac{301-H}{300}+7\times \frac{301-L}{300}+9\times \frac{301-C}{300}$$

where IS is between 0 and 21.

### 2.8. Evaluation of Anti-Irritative Potential

The eggs were prepared as mentioned above, and the method previously described [26] was adapted to our laboratory conditions. To determine the anti-irritative effect of aspartame, the highest concentration tested in ovo (30 mM) was applied in a volume of 600 µL. In parallel, a solution of dexamethasone (DEX), known for its anti-irritative effect, was applied in the same volume. After 3 h of incubation, 300 µL of 0.5% sodium dodecyl sulfate (SDS) in H_2_O were applied to the eggs. Vascular effects (lysis, stasis, and hemorrhage) were observed under a microscope for 5 min.

At the end of the experiment, the following parameters were calculated:

(4) $$H_{\mathrm{AI}}=\frac{H}{H_{\mathrm{SDS}}}$$

H_AI_ = hemorrhage time after pretreatment with ASP/DEX and H_SDS_ = 0.5% SDS addition/hemorrhage time without pretreatment with ASP/DEX

(5) $$L_{\mathrm{AI}}=\frac{L}{L_{\mathrm{SDS}}}v$$

L_AI_ = vascular lysis time after pretreatment with ASP/DEX and L_SDS_ = 0.5% SDS addition/hemorrhage time without pretreatment with ASP/DEX

(6) $$C_{\mathrm{AI}}=\frac{C}{C_{\mathrm{SDS}}}$$

C_AI_ = vascular coagulation time after pretreatment with ASP/DEX and C_SDS_ = 0.5% SDS addition/hemorrhage time without pretreatment with ASP/DEX.

### 2.9. Statistical Analysis

The statistical interpretation of the data was performed with GraphPad Prism version 8.0.0 for Windows, GraphPad Software, San Diego, California USA, www.graphpad.com. The results were expressed as the mean ± standard deviation (SD). One-way ANOVA statistical test was applied to identify the statistical differences between the groups (control and treated), followed by Dunnett’s post-test (** *p* < 0.01, *** *p* < 0.001, and **** *p* < 0.0001).

## 3. Results

### 3.1. Aspartame Induced Cytotoxicity in a Concentration-Dependent Manner

**Figure 1:**
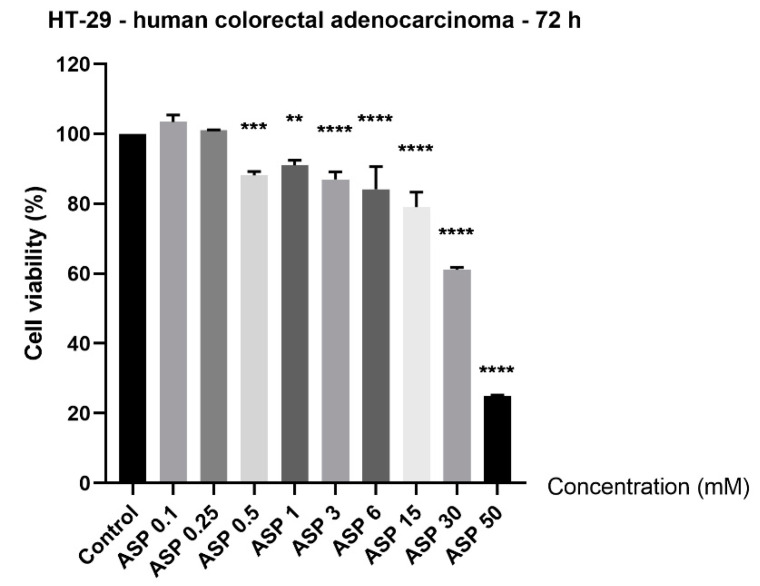
Assessment of the in vitro effect of aspartame (ASP; 0.1, 0.25, 0.5, 1, 3, 6, 15, 30, and 50 mM) on HT-29 cell viability after a 72 h treatment by applying the Alamar blue assay. The results are presented as cell viability percentage (%) normalized to control (PBS-stimulated) cells and are expressed as mean values ± SD of three independent experiments performed in triplicate. To identify the statistical differences between the control and the treated group, one-way ANOVA analysis was conducted, followed by Dunnett’s multiple comparisons post-test (** *p* < 0.01, *** *p* < 0.001, and **** *p* < 0.0001).

Since the role of aspartame in colorectal cancer is controversial, in the present study, its impact on HT-29 cell viability was verified by incubating the cells with ascending concentrations (0.1, 0.25, 0.5, 1, 3, 6, 15, 30, and 50 mM) for 72 h. The results indicated signs of cytotoxicity in HT-29 cells starting with the concentration of 0.5 mM, but the most significant decrease in the percentage of viable cells was observed at the highest concentrations tested (15, 30, and 50 mM): 79.35%, 61.20%, and 25.01%, respectively (Figure 1). The lowest concentrations tested—0.1 and 0.25 mM—did not affect HT-29 cell viability; moreover, it could be said that a slight stimulatory effect was noticed (Figure 1). The calculated half maximal inhibitory concentration (IC_50_) was 15.82 mM. The obtained results were normalized to control cells. Control cells were considered the cells stimulated with PBS only, being the solvent used for aspartame (at the same concentrations tested for ASP: 0.1, 0.25, 0.5, 1, 3, 6, 15, 30, and 50 mM).

### 3.2. High Concentrations of Aspartame Changed HT-29 Cells’ Morphology and Shape

**Figure 2:**
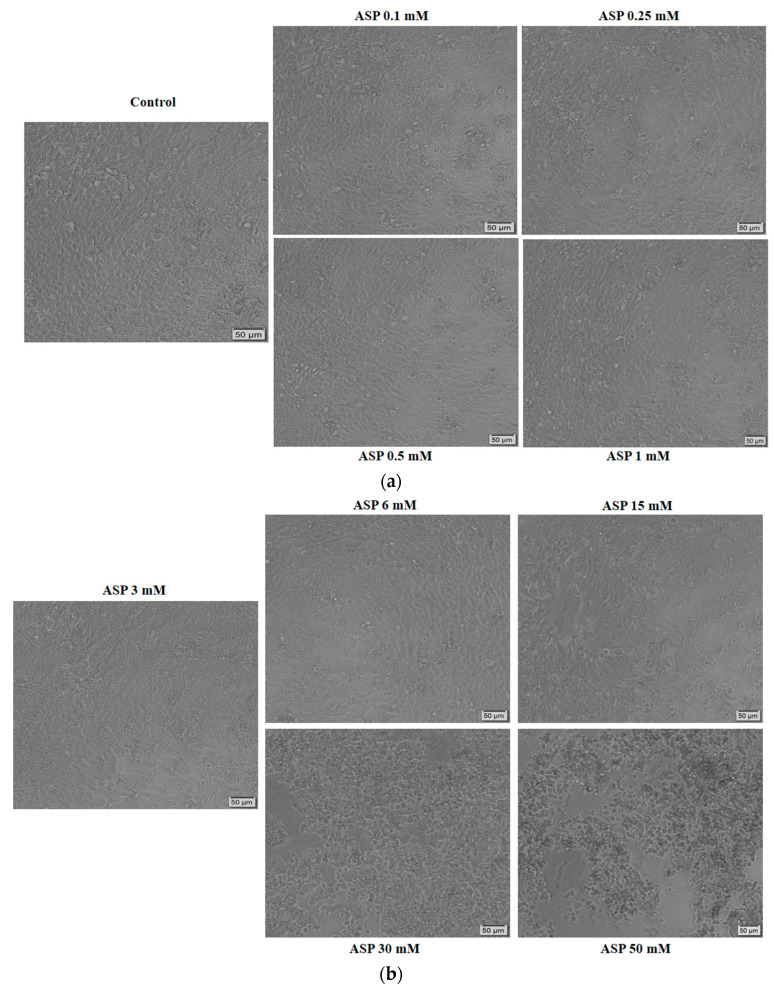
(**a**). Morphological changes induced by aspartame solution in HT-29 cells: Control (non-stimulated cells) and cells stimulated with increasing concentrations (0.1, 0.25, 0.5, and 1 mM) of aspartame (ASP) in PBS (phosphate-buffered saline) for 72 h. The pictures were taken at 72 h post-stimulation. The scale bars represent 50 μm. (**b**) Morphological changes induced by aspartame solution in HT-29 cells: Cells stimulated with increasing concentrations (3, 6, 15, 30, and 50 mM) of aspartame (ASP) in PBS for 72 h. The pictures were taken at 72 h post-stimulation. The scale bars represent 50 μm.

To determine the cytotoxic impact of ASP in terms of cellular morphological changes, the HT-29 cells were monitored and photographed at 72 h post-treatment (Figure 2a,b). The lowest concentrations of ASP (0.1, 0.25, 0.5, 1, 3, and 6 mM) did not alter the HT-29 cells’ confluence, their capacity to adhere to the culture plate, or their shape; and some round and floating cells were detected in the wells stimulated with 1, 3, and 6 mM ASP. As for the HT-29 cells stimulated with 15, 30, and 50 mM ASP, significant changes were noticed: a decreased confluence as compared to control cells, multiple round cells that were floating, and cellular debris (at 50 mM) (Figure 2b). These data endorse the cell viability results and confirm the cytotoxicity of ASP in HT-29 cells at high concentrations. It was also verified the impact of PBS—the solvent of ASP—at the same concentrations (0.1, 0.25, 0.5, 1, 3, 6, 15, 30, and 50 mM), and no significant morphological changes were detected as compared to control cells (unstimulated).

### 3.3. Cell Migration

**Figure 3:**
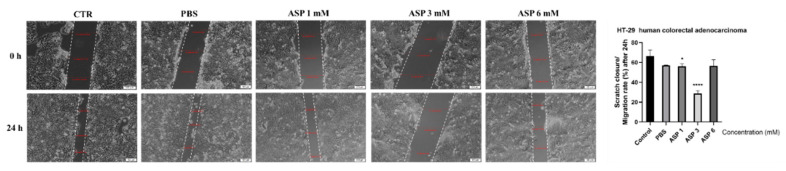
The effect of ASP solution (1, 3, and 6 mM) on HT-29 cell migratory capacity following 24 h of stimulation. Scratch widths were pictured at 0 and 24 h post-exposure. Scale bars represent 100 μm. The results are expressed as scratch closure/migration rate (%) at 24 h post-stimulation. The data represent the mean values ± SD of three independent experiments. One-way ANOVA analysis was applied to determine the statistical differences, followed by Dunnett’s multiple comparisons post-test (* *p* < 0.05 and **** *p* < 0.0001).

To determine the impact of ASP solution on HT-29 cell migratory capacity (a specific feature of cancer cells) the scratch assay protocol was applied. For this experiment, three concentrations were selected—1, 3, and 6 mM—concentrations that did not reduce significantly the HT-29 cells’ viability. The control cells (unstimulated) presented the highest rate of migration, followed by the cells stimulated with PBS (Figure 3). A significant inhibition of cells migration was measured for the cells stimulated with ASP 3 mM (Figure 3), whereas in the case of 1 and 6 mM, the inhibitory effect was not significant.

### 3.4. Angiogenic Effect

**Figure 4:**
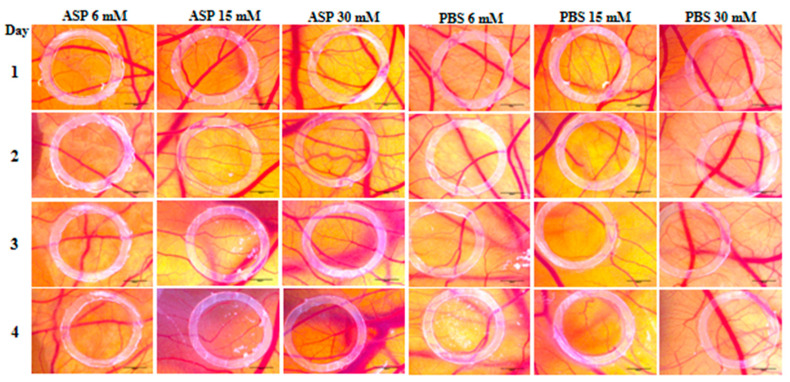
Microscopic images of the effects of ASP (6, 15, and 30 mM) and PBS (6, 15, and 30 mM) on normal angiogenesis after 4 days of treatment. Scale bars represent 500 μm.

The assessment of ASP effect on angiogenesis was performed for four days. On the first day after ASP addition, no noticeable change in angiogenesis was recorded inside the ring, the aspect of blood vessels being similar to that outside the ring where the test solution was not applied. The first changes in angiogenesis were observed on the second day of after ASP application, mainly in the case of the higher concentrations of 15 and 30 mM (Figure 4). On the third day, the 15 mM ASP solution induced thickened vascular pathways based on branches obtained by angiogenesis by intussusception and sprouting, thus indicating a pro-angiogenic action. At the 30 mM concentration, a multitude of interconnected vessels were observed, also suggesting a stimulated angiogenic reaction. On the last day of observation, the strongest stimulating effect on angiogenesis was observed at the concentration of 15 mM ASP. In this case, thickened and strongly branched vacuums were present. Also, a modification of the vascular architecture with the formation of wide meshes was observed. In the case of the 30 mM concentration, an average effect of stimulating angiogenesis was noticed, whereas the 6 mM concentration had no impact on the formation of blood vessels. In the case of the PBS control solution, a normal angiogenesis process was observed, with the aspect of blood vessels inside of the ring being comparable to the outside one (Figure 4).

### 3.5. Irritant Potential

**Figure 5:**
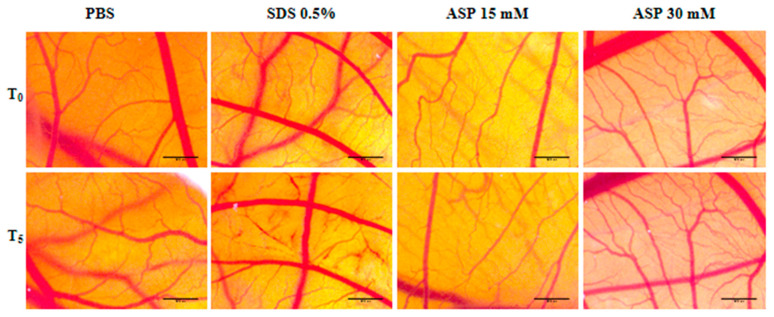
Assessment of the irritant potential of ASP by the hen’s egg test on chorioallantoic membrane (HET-CAM) method, where: T_0_—0 min after application of test compounds time; T_5_—at 5 min after application of test compounds. Stereomicroscopic images of CAMs (Chorioallantoic Membrane Assay) inoculated with SDS, the positive control; PBS, the solvent used to solubilize ASP; and ASP, the test compound, at 15 and 30 mM. Scale bars represent 500 μm.

To test the irritant potential, two concentrations of ASP (15 and 30 mM) and PBS (control) were applied. In the case of the 15 mM ASP solution, a slight punctiform hemorrhage was observed at 2 min and 3 s after the application (Figure 5). In the case of the 30 mM ASP solution, a slight dilation of the blood vessels was observed at 1 min and 56 s, accompanied by an increase in the speed of blood circulation in the vessels. In the case of the PBS control solution, no change was observed in the 5 min after application of a volume of 600 µL. Regarding the positive control (SDS), the following effects could be observed: coagulation at 14 s, lysis at 44 s, and hemorrhage at 55 s after application (Table 1).

### 3.6. Anti-Irritant Potential

**Figure 6:**
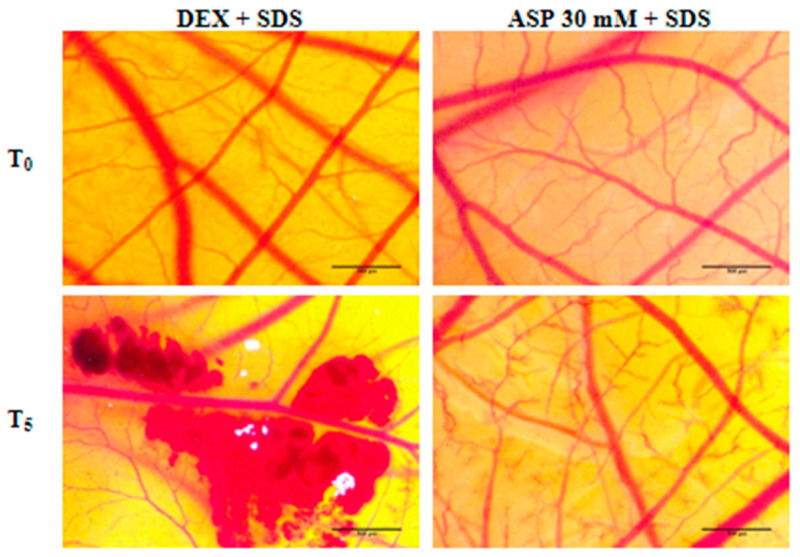
Assessment of the potential anti-irritant effect of ASP. Stereomicroscopic images of CAMs treated with 30 mM ASP and DEX, followed by irritation with SDS. Scale bars represent 500 μm.

Table 2 shows the irritability scores of 30 mM ASP and dexamethasone and the times of occurrence of vascular effects (hemorrhage, lysis, and vascular stasis) before (H_DEX_, L_DEX_, C_DEX_, H_ASP_, L_ASP_, C_ASP_) and after the treatment with 0.5% SDS (H_AI_, L_AI_, C_AI_). In the case of treatment with DEX, there is an absence of anti-irritant effect, while treatment with 30 mM ASP causes a reduction in IS, exerting a moderate anti-irritant effect (Figure 6).

## 4. Discussion

In recent years, several safety concerns regarding the use of aspartame have been raised, and even an association with the risk of carcinogenesis (of brain, digestive, hematopoietic, and reproductive systems) has been suggested, but the available scientific data concerning this issue are inconclusive and contradictory. To this end, in the present study, the potential role of aspartame in colorectal cancer was investigated by verifying its cytotoxic effects in vitro on HT-29 human colorectal carcinoma cells and its angiogenic potential in ovo by means of the CAM assay.

Europe’s population consumes more than 2000 tons of ASP annually as an artificial sweetener in various foods [27]. ASP has been an FDA-approved sweetener (Food and Drug Administration) since 1981 (46 FR 38283), initially only for uses under specific conditions or in dry bases for particular foods, but in 1996, the organization approved it as a “general purpose sweetener” [28]. The acceptable daily intake (ADI) for aspartame was established at 50 mg/kg by the FDA in 2005 and at 40 mg/kg by the EFSA (European Food Safety Authority) in 2006 [29]. Currently, ASP is incorporated into a large variety of foods such as desserts, yoghurt, chewable multivitamins, breakfast cereals, and soft drinks, as well as in many pharmaceutical products, making it available to millions of people worldwide [6]. Structurally, ASP is a synthetic dipeptide of aspartyl–phenylalanine methyl ester [3,9], which is completely metabolized to its main components after intake [3]. The classification of aspartame as a semi-synthetic peptide is due to its chemistry involving the amino acids phenylalanine and aspartate connected by a methanolic column. The difficulty of classifying it as a non-nutritive or nutritive compound derives from the metabolic processes involved, not being a strictly non-caloric compound [30].

The wide application of aspartame in a plethora of products for human use and its increased consumption led to numerous studies that were focused on the side effects and toxicity of ASP. Regarding the use of ASP in toxic doses, it has been observed that it causes alterations in the homeostasis of the cell membrane, leading to the appearance of oxidative stress at various levels of the brain such as the cerebral cortex, cerebellum, or hippocampus. Behavioral changes such as increased anxiety and histopathological changes in different regions of the brain were also recorded [31]. At a dose of 1000 mg/kg, liver damage was noticed by altering the antioxidant system via the glutathione-dependent system [32]. Administration of aspartame in low doses, below 40 mg/kg/day, was associated with an increase in oxidative stress in the liver [33], kidney [34], and at the cardiac level [35]. Regarding the risk of carcinogenesis related to ASP consumption, the opinions are divided: the epidemiological studies results do not assert a direct link between consumption of low- and no-calorie sweeteners (aspartame) and an augmented risk of developing cancer in humans [7], whereas several animal studies indicated a high incidence of cancers dose-dependent, including hematological tumors [36]. The connection of ASP with colorectal cancer is also debatable. A case control study developed by Mahfouz [37] and collaborators in 2014 included the intake of artificial sweeteners as the third risk factor of colorectal carcinoma [37]. A more recent study conducted in 2018 by Guercio et al. observed that an increased consumption of artificially sweetened beverages could be associated with a markedly decreased cancer recurrence and death in patients diagnosed with stage III colon cancer [19].

The toxicological profile of ASP portrayed based on the outcomes of the literature urged a novel toxicological assessment of aspartame by the EFSA. The final report of EFSA (released December 2013), which comprised the analysis of the in vitro, in vivo and clinical studies concerning ASP conducted up to 2011, concluded that “there were no safety concerns at the current ADI of 40 mg/kg bw/day. Therefore, there was no reason to revise the ADI for aspartame” [38,39]. Another aspect mentioned in the report was the estimated ASP daily intake values (data acquisition from 26 studies conducted in 17 European countries), presented as the mean (1.2 to 5.3 mg/kg/day) and highest values (1.9 to 15.6 mg/kg/day), indicating that these values represent less than 50% of the ADI and the risk of toxicity is low [40,41]. Aspartame intake occurs exclusively by oral ingestion and is metabolized to three main metabolites (methanol—10%, phenylalanine—50%, and aspartic acid—40%) via enzymatic hydrolysis in the gastrointestinal lumen and in the internal intestinal mucosa cells, so ASP “per se” does not enter the general circulation [41,42]. Of interest was also the impact of ASP on the gut microbiome, since the gastrointestinal lumen represents the site of metabolization for ASP and where ASP can exert its effect as intact molecule [43,44,45]. Still, based on the data presented in the literature, a direct connection between ASP’s effect and detrimental effects on gut microbiota cannot be stated, and further studies are required [42]. However, a direct link was described between gut microbiome status and colorectal cancer development [46,47].

In recent years, a novel in vitro approach was applied to investigate ASP as intact molecule by using immortalized cancer cell lines (HT-29, Caco-2, PC12, HeLa cells) as experimental models [17,48,49,50], but the data are still scarce in this research direction. The cell lines used for the in vitro models are considered representative cell lines since most of them are selected based on having morphological characteristics that are similar to the in vivo equivalent and thus generate important data regarding the safety and efficacy of a test compound.

The cell line selected in the present study for the in vitro approach was HT-29, the human colorectal carcinoma cell line. The HT-29 cell line was the first established (1964) colon carcinoma cell line of human origin and was used as the cell culture model in the studies regarding the biology of human colorectal cancers. Recently, HT-29 cells have been employed in studies regarding the intestinal cells’ function due to the following characteristics: (i) the cells express functional receptors for hormones and peptides, (ii) they can synthesize the receptor of dimeric immunoglobulin A; and (iii) they can be differentiated in culture under the impact of differentiation inducers (sodium butyrate, dimethyl sulfoxide, etc.). Moreover, these cells possess the capacity to express features of enterocytes and mucus-producing cells (the mature intestinal cells) and to secrete metabolites, growth factors, pro-angiogenic factors (IL-15 and vascular endothelial growth factor (VEGF)), cytokines, and other factors that sustain cellular survival [51,52]. It was also proved that HT-29 cells maintain their cellular properties unchanged even after 100 passages [52]. Based on the abovementioned arguments, in this study, the HT-29 cell line was applied as the model for studying the biology of colorectal cancer.

The concentrations tested in the study (0.1, 0.25, 0.5, 1, 3, 6, 15, 30, and 50 mM, and their equivalents in mg: 0.02943, 0.07357, 0.14715, 0.2943, 0.8829, 1.7658, 4.4145, 8.829, and 14.715 mg) were selected based on a thorough survey of the literature regarding aspartame doses tested in vitro [17,48,49,50]. Moreover, were taken into consideration the ADI value for ASP, of 40 mg/kg/d, and the mean and highest estimated values for ASP daily intake: 1.2 to 5.3 mg/kg/d and 1.9 to 15.6 mg/kg/d, respectively. We performed an estimative calculation for the equivalent in vivo doses of the in vitro concentrations by applying the formulas described by Levy [53], and the estimative calculated dose related to the IC_50_ of 4.67 mg obtained was lower than the ADI.

The results obtained in this study showed a dose-dependent cytotoxic effect of ASP on HT-29 cells; the most significant decrease in percentage of viable cells was recorded at the highest concentrations tested, of 15, 30, and 50 mM (Figure 1). The lowest concentrations tested (0.1 and 0.25 mM ASP) induced a slight increase in HT-29 cell viability (Figure 1). These data were confirmed by the morphological changes observed in the cells treated with 15, 30, and 50 mM, namely reduced confluence, round and detached cells, and cellular debris, which represent specific signs of cytotoxicity (Figure 2b).

Similar results to our findings were described by van Eyk, who studied the effect of five artificial sweeteners, including aspartame, on Caco-2 and HT-29 colorectal adenocarcinomas. In both cases, the low concentrations of ASP induced a slightly higher cell viability, while exposure to concentrations as high as 10 mM reduced the viability percentages in a dose-dependent manner. The greatest effect was observed after the 72 h treatment. The HT-29 cell line seemed to be more sensitive to the activity of all artificial sweeteners, aspartame included [17]. Pandurangan et al. investigated the effects of aspartame on human cervical carcinoma cells (HeLa) and reported that cell viability significantly declined after 48 h of stimulation with 10 µM, 100 µM, 1 mM, 10 mM, and 20 mM ASP. Regarding its molecular mechanism, fluorescence microscopy revealed that the exposure to ASP resulted in the fragmentation of nuclei, as well as chromatin condensation and the appearance of apoptotic bodies. Severe DNA damage has only been observed at high concentrations of 10 and 20 mM [50]. Another study highlighted that ASP induces significant changes in the mRNA expression of apoptotic genes in HeLa cells, down-regulating the expression of the tumor suppressor gene p53 and the apoptotic gene bax, while up-regulating the expression of bcl-2, which suggests that ASP manifests a cytotoxic effect via apoptosis [9]. Another study found an absence of significant cytotoxic effect in Caco-2 cells after exposure to 0.1–0.5 mM ASP for 24 h, and neither apoptotic nor necrotic cells were noticed [48]. These data are in agreement with our results, which showed a cytotoxic effect of ASP only at the highest concentrations tested: 15, 30, and 50 mM after 72 h of treatment. Another aspect that was investigated in this study is the impact of ASP (1, 3, and 6 mM) on HT-29 cell migratory capacity. The obtained data showed an inhibition of migration at 3 mM, whereas at 1 and 6 mM, ASP did not affect cell migration (Figure 3). Based on these results, the pattern of ASP regarding HT-29 cell migration could be considered undefined.

An inhibitory effect of 0.1 mM ASP was detected in Caco-2 cells, thus interfering in the wound healing process [48]. A recent study proved that ASP (3.125–100 mg/L) exerted a dose-dependent moderate cytotoxic effect after 48 h in human blood cells, and no significant effect was recorded in the antioxidant/oxidative balance. Moreover, ASP proved to induce genotoxicity in human blood cells after 72 h at an IC_50_ of 287.342 mg/L [5]. The lack of toxic effects of ASP at low concentrations was also shown in a recent study conducted on human umbilical vein endothelial cells stimulated with concentrations between 0.01 and 1 mM for 24, 48, 72, and 96 h [54].

Taking into consideration the idea elaborated by previous studies that ASP has a pro-angiogenic effect, this aspect was also assessed in the present study. Angiogenesis is the process of forming new blood vessels and has implications for many diseases such as cancer, psoriasis, and chronic inflammatory diseases [55]. The CAM assay is one of the most widespread in vivo methods for analyzing the effects of various substances on angiogenesis [24]. In the present study, we aimed to test the effect of aspartame on angiogenesis using the CAM method. Three different concentrations of ASP (6, 15, and 30 mM) were tested. It has been observed that ASP has a strong pro-angiogenic effect at concentrations of 15 and 30 mM, concentrations that are below the daily intake value of aspartame (40 mg/kg) established by the European Food Safety Authority (EFSA) [56].

Corresponding results were presented by Yesildal et al. [20]. Doses of ASP of up to 60 mM were tested on CAM, noting that ASP stimulates angiogenesis in a dose-dependent manner. The pro-angiogenic effect of aspartame was tested both on CAM and on a mouse model, noting that ASP also has applications in wound healing. The effects of ASP on the placenta were studied in vivo using adult white rats. It has been observed that ASP causes a significant increase in vascular endothelial growth factor (VEGF), which has implications for the process of angiogenesis in pregnancy [57]. Another in vitro study on endothelial cells and fibroblasts showed that ASP increases the production of reactive oxygen species (ROS) associated with the cytotoxic effect, increases the level of the inflammatory mediator IL-6, and has a pro-angiogenic effect at low doses by stimulating regenerative cytokine production and activation of the MAPK pathway [58].

The data obtained from applying the HET-CAM assay to evaluate the irritant potential of ASP classify 15 and 30 mM ASP as weak irritant, according to the Luepke score [59]. Moreover, when compared to dexamethasone, known as an anti-irritative compound, ASP proved to be less irritant by reducing the vascular effects of SDS (hemorrhage, lysis, and vascular stasis) (Figure 6, Table 2).

## 5. Conclusions

In conclusion, the findings of the present study indicate a cytotoxic effect of aspartame at high concentrations (15, 30, and 50 mM) in HT-29 cells, characterized by a decreased percentage of viable cells and morphological changes (apoptotic signs). Moreover, the concentrations that proved to be cytotoxic in vitro (15 and 30 mM) exerted a pro-angiogenic effect in ovo and presented a weak irritant potential at vascular level. These data might represent a background for further studies aiming to elucidate aspartame’s mechanism of action in tumor cells.

## Figures and Tables

**Table 1:** Irritation score (IS) for SDS (sodium dodecyl sulfate), PBS, and ASP at 15 mM and 30 mM, and the occurrence time of hemorrhage (tH), lysis (tL), and coagulation (tC).

	SDS 1%	PBS	ASP 15 mM	ASP 30 mM
IS	18.7	0.07	3.02	3.13
tH	55 s	300 s	123 s	116 s
tL	44 s	300 s	300 s	300 s
tC	14 s	300 s	300 s	300 s

**Table 2:** Anti-irritant effect of 30 mM ASP and dexamethasone (DEX) after the irritation of CAM with 0.5% SDS (H_AI_ = hemorrhage time after pretreatment with ASP/DEX; L_AI_ = vascular lysis time after pretreatment with ASP/DEX; C_AI_ = vascular coagulation time after pretreatment with ASP/DEX).

	SDS 0.5%	DEX + SDS	ASP 30 mM + SDS
IS	18.68	18.48	17.57
tH	55 s	25 s	60 s
tL	44 s	35 s	58 s
tC	14 s	45 s	38 s
H_AI_		0.98	1.09
L_AI_		1.47	1.31
C_AI_		3.21	2.71
